# HSP70 protects H9C2 cells from hypoxia and reoxygenation injury through STIM1/IP3R

**DOI:** 10.1007/s12192-022-01290-0

**Published:** 2022-07-16

**Authors:** TianYu Liu, Zhaodong Juan, Bin Xia, GuanHua Ren, Zhen Xi, JunWen Hao, ZhongDong Sun

**Affiliations:** 1 The First Affiliated Hospital of Weifang Medical University, Weifang People’s Hospital Cardiovascular Surgery, Weifang, 261000 China; 2 Shandong Provincial Medicine and Health Key Laboratory of Clinical Anesthesia, School of Anesthesiology, Weifang Medical University, Weifang, 261000 China; 3 School of Clinical Medicine, Weifang Medical University, Weifang, 261000 China

**Keywords:** Myocardial ischemia–reperfusion injury, Heat shock protein 70, Glucose deprivation, Oxygen deprivation, Stromal interaction molecule 1, Cell apoptosis, Inositol 1,4,5-triphosphate receptor

## Abstract

Hypoxia/reoxygenation (H/R) is used as an in vivo model of ischemia/reperfusion injury, and myocardial ischemia can lead to heart disease. Calcium overload is an important factor in myocardial ischemia–reperfusion injury and can lead to apoptosis of myocardial cells. Therefore, it is of great clinical importance to find ways to regulate calcium overload and reduce apoptosis of myocardial cells, and thus alleviate myocardial ischemia–reperfusion injury. There is evidence that heat shock protein 70 (HSP70) has a protective effect on the myocardium, but the exact mechanism of this effect is not completely understood. Stromal interaction molecule 1 and inositol 1,4,5-triphosphate receptor (STIM/1IP3R) play an important role in myocardial ischemia–reperfusion injury. Therefore, this study aimed to investigate whether HSP70 plays an anti-apoptotic role in H9C2 cardiomyocytes by regulating the calcium overload pathway through STIM1/IP3R. Rat H9C2 cells were subjected to transient oxygen and glucose deprivation (incubated in glucose-free medium and hypoxia for 6 h) followed by re-exposure to glucose and reoxygenation (incubated in high glucose medium and reoxygenation for 4 h) to simulate myocardial ischemia reperfusion-induced cell injury. H9C2 cell viability was significantly decreased, and lactate dehydrogenase (LDH) release and apoptosis were significantly increased after oxygen and glucose deprivation. Transfection of HSP70 into H9C2 cells could reduce the corresponding effect, increase cell viability and anti-apoptotic signal pathway, and reduce the apoptotic rate and pro-apoptotic signal pathway. After hypoxia and reoxygenation, the expression of STIM1/IP3R and intracellular calcium concentration of HSP70-overexpressed H9C2 cells were significantly lower than those of hypoxia cells. Similarly, direct silencing of STIM1 by siRNA significantly increased cell viability and expression of anti-apoptotic protein Bcl-2 and decreased apoptosis rate and expression of pro-apoptotic protein BAX. These data are consistent with HSP70 overexpression. These results suggest that HSP70 abrogates intracellular calcium overload by inhibiting upregulation of STIM1/IP3R expression, thus reducing apoptosis in H9C2 cells and playing a protective role in cardiomyocytes.

## Introduction

H/R is used as an in vivo model of ischemia/reperfusion injury, and myocardial ischemia can lead to heart disease. Myocardial ischemia–reperfusion injury (MIRI) can occur with partial or complete blockage of the coronary arteries that supply blood to the myocardium, resulting in ischemia and hypoxic injury. Even if the vascular obstruction is quickly resolved and the ischemic heart muscle is resupplied with blood, tissue damage can be further aggravated by reperfusion (Wu et al. 2018). The mechanisms involved in MIRI are complex and include intracellular calcium ion overload, increased mitochondrial permeability (Morciano et al. 2017), oxidative stress (Venardos et al. 2007), and inflammatory response (Eltzschig and Eckle 2011). These involve a variety of signaling pathways and molecular mechanisms, and therefore, there is still no effective method to prevent and treat MIRI. Recently, a growing number of studies have shown that calcium overload plays a key role in MIRI damage (Wang et al. 2021). Calcium overload during MIRI is caused by extracellular calcium ions flowing into the cell through calcium ion channels and Na^+^/Ca^2+^ exchangers. This leads to an abnormal increase in intracellular calcium ions, activation of protein kinases, rupture of cell membranes, and finally apoptosis of the cell (Yue et al. 2017). Despite this, there is still no effective treatment to prevent MIRI caused by calcium overload. This study aimed to find potential drug targets that might prevent calcium overload in MIRI and therefore improve the prognosis of patients.

Stromal interaction molecule 1 (STIM1) is a single transmembrane endoplasmic reticulum protein. It consists of an EF-sterile α-motif complex, a Ca^2+^ binding site in the ER lumen, a cytoplasmic C-terminal region containing three helically coiled segments (CC1, CC2, and CC3), and a flexible region (Tiffner et al. 2021). STIM1 is one of the key proteins in store-operated calcium entry (SOCE) (Rosenberg et al. 2021). When the endoplasmic reticulum (Er) calcium reservoir is depleted, STIM1 can rapidly accumulate and shift to the region opposite the cell membrane, which activates ORAI (Sather and Dittmer 2019; Michelucci et al. 2018), resulting in a large number of extracellular calcium ions flowing into the cell (Silva-Rojas et al. 2020). Inositol 1,4,5-triphosphate receptor (IP3R) is an important calcium ion channel that regulates Ca^2+^ flow between the endoplasmic reticulum, other intracellular organelles, and the cytoplasm (Hamada and Mikoshiba 2020). IP3R is present in all cell types, is composed of the key IP3 binding domain, inhibitory binding domain, channel domain, and regulatory/coupling domain, and can only be activated in the presence of calcium ions (Ahumada-Castro et al. 2021). Moreover, when IP3R is activated and calcium ions in the endoplasmic reticulum are depleted, IP3R will actively bind with and activate STIM1 to trigger SOCE, resulting in a large influx of extracellular calcium ions to maintain the intracellular calcium ion concentration (Sampieri et al. 2018).

As one of the most conserved proteins (Mayer and Bukau 2005), HSP70 is ubiquitous in a wide range of organisms (Rosenzweig et al. 2019). HSP70 is composed of a substrate-binding domain and a nucleotide-binding domain (Mayer and Gierasch 2019). HSP70 binds and hydrolyzes ATP, inducing a conformational change that produces corresponding biological effects (Kampinga et al. 2010). HSP70 mediates the folding of newly synthesized proteins, prevents the aggregation and refolding of misfolded denatured proteins (Larburu et al. 2020), regulates protein activity, transfers peptides to the appropriate organelles (e.g., endoplasmic reticulum and mitochondria), and breaks down protein complexes to enable cells to cope with severe external conditions (Fernández-Fernández and Valpuesta 2018). HSP70 exerts anti-apoptotic effects by targeting different intrinsic and extrinsic pathways of apoptosis (Roufayel and Kadry 2019). For example, knocking out HSP70 in tumor cells can effectively inhibit tumor development (Kumar et al. 2016; Albakova et al. 2020). We have demonstrated that HSP70 also plays an important role in combating myocardial apoptosis, but the exact mechanism remains unclear. We hypothesized that HSP70 inhibits one or more calcium channels, thereby attenuating the calcium overload that occurs in MIRI and thus playing an anti-apoptotic role. The purpose of this study was to investigate the regulatory effect of HSP70 on hypoxia-reoxygenation (H/R)-induced apoptosis of cardiomyocytes and to elucidate the corresponding molecular mechanism.

## Materials and methods

### Cell culture and treatment

H9C2 cells (ProCell, Wuhan, China) were subcloned from BD1X rat embryonic heart tissue and cultured in Dulbecco’s modified Eagle medium supplemented with 10% fetal bovine serum and 1% penicillin/streptomycin double-antibody. The culture environment was 37 ℃ in an incubator with 5% CO_2_ and normal ambient oxygen levels. To simulate injury from oxygen and glucose deprivation, the cells are first replaced with a glucose-free medium without fetal bovine serum and double-antibody and then placed in a three-gas incubator with an internal environment of 1% O2, 5% CO2, 94% N2, 37 ℃ for 6 h. Then they were reoxygenated by incubation in a normal oxygen environment, with high glucose DMEM medium, for 4 h.

### Cell transfection

The amplified HSP70 (the HSP70 gene was transfected into rat-derived H9C2 cells, and the transfected gene was queried as HSPa1b on the official NCBI website) were ligated into pcDNA3.1 vector (Shanghai, China) to obtain pcDNA3.1/HSP70 with the empty vector as control. The small interfering RNA (siRNA) to silence HSP70, and its negative control, and the small interfering RNA (siRNA) to silence STIM1, and its negative control, were synthesized by GenePharma (Shanghai, China). The cells were transfected with Lipofectamine 3000 reagent (Thermo Fisher Scientific, MA, USA), and the plasmid and siRNA were delivered into the H9C2 cells. The transfection was conducted over 24 h before subsequent oxygen and glucose deprivation assays. There were eight experimental groups in this study: control, HR, HR + HSP70, HR + NC (HSP70), HR + siRNA (HSP70), HR + NC (siRNA), HR + siRNA (STIM1), and HR + NC (STIM1).

The transfected siRNA sequence was as follows: siRNA (STIM1): sense (5′-3′) GCUGCUGGUUUGCCUAUAUTT; antisense (5′-3′) AUAUAGGCAAACCAGCAGCTT; siRNA (HSP70): sense (5′-3′) GACCUGAACAAGAGCAUCATT; antisense (5′-3′) UGAUGCUCUUGUUCAGGUCTT.

### Flow cytometry

The FITC-coupled Annexin V Apoptosis Detection Kit was used in this study (Becton, Dickinson and Company, USA). Adherent cells were digested with EDTA-free trypsin and centrifuged at 1800 rpm for 8 min. The supernatant was discarded and the precipitate was cleaned with PBS for 8 min at 1800 rpm. The supernatant was discarded, 500 µl annexin binding buffer was added to resuspend the cells, and this suspension was incubated with 5 µl annexin V/FITC at room temperature for 15–20 min in the dark. A 5 µl propidium iodide was added before flow cytometry was performed. Fluorescence was detected by a flow cytometer (BD Biosciences), and cell apoptosis was calculated using NovoExpress software (ACEA Biosciences Inc, Hangzhou, China). Apoptosis was calculated as the sum of early apoptosis and late apoptosis.

### Cell counting kit-8 (CCK-8) assay

After transfection and H/R treatment, the CCK-8 assay (BioSharp, Hefei, China) was used to detect cell viability. The transfected and treated H9C2 cells were cultured in a 96-well plate supplemented with a 10 µl CCK‐8 solution. After 1 h of co-culture at 37 °C, the absorbance was assessed at 450 nm using a microplate reader (Bio-Rad, Hercules, CA, US).

### LDH release

The destruction of cell membrane structure caused by apoptosis will lead to the release of enzymes in cytoplasm into culture medium, including LDH with relatively stable enzyme activity. By detecting the LDH activity released from cytoplasm into culture medium, the cytotoxicity can be quantitatively analyzed. Lactate dehydrogenase (LDH) release was detected using the Lactate Release Test Kit (Biotech, Shanghai, China) to evaluate the integrity of the hypoxic posterior membrane of H9C2 cells and cytotoxicity. H9C2 cells are incubated in a 96-well plate, the supernatant is absorbed after the hypoxic reoxygenation model is established, the supernatant is extracted after low centrifugation and mixed with the LDH reagent, and the data is read by a microplate reader. The result is calculated according to the cytotoxic formula in the instructions: cytotoxicity (%) = (treatment sample absorbance − sample control well absorbance) / (absorbance of the maximum enzyme activity of the cell − sample control well absorbance).

### Laser confocal microscopy

To measure intracellular calcium concentration, H9C2 cardiomyocytes were incubated with FluO-3/AM (Alexis, Invitrogen, USA) at 37 ℃ for 30 min. Fluorescence was monitored at 528 nm using an LSM510 laser confocal scanning microscope. The image was obtained by NIS-Elements Viewer software, and the fluorescence intensity of the image was quantitatively analyzed by ImageJ software.

### qRT-PCR

Total RNA was extracted with TRIzol reagent (Thermo Fisher Scientific, Shanghai, China). Reverse transcription was performed with a HiScript III All-in-One RT SuperMix Perfect for qPCR (Vazyme, Nanjing, China). The primer sequences were as follows: STIM1, forward, 5′-TGGAGCTGCCACAGTATGAG-3′ and reverse, 5′-TGATTGTGGCGAGTCAAGAG-3′; IP3R, forward, 5′-CATTTGCCGACTCTGCTACA-3′ and reverse, 5′-TGTGCTTTTCCAGGAGCTTT-3′; BAX, forward, 5′-CAGGCGAATTGGCGATGAAC-3′ and reverse, 5′-GGGTCCCGAAGTAGGAAAGG-3′; BCL-2, forward, 5′-AGCATGCGACCTCTGTTTGA-3′ and reverse, 5′-TCACTTGTGGCCCAGGTATG-3′, GAPDH, forward, 5′-GTTACCAGGGCTGCCTTCTC-3′ and reverse, 5′-ACCAGCTTCCCATTCTCAGC-3′. The synthesized cDNA products were analyzed by real-time PCR using 2 × ChamQ SYBR qPCR Master Mix. GAPDH was used as an internal reference.

### Western blot

After transfection and treatment, the cells were collected and lysed in RIPA buffer (Solarbio, Beijing, China) containing PMSF (Solarbio, Beijing, China) buffer. Protein was extracted and electrophoresed on SDS–PAGE gels and transferred to PVDF membranes (Millipore Corp., Bedford, MA, USA). The membranes were blocked with TBST buffer containing 5% skim milk at room temperature for 2 h and then incubated overnight at 4 ℃ with specific antibodies against STIM1 (1:5000; Abcam, AB_2197884, Cambridge, UK), BAX (1:1,000; Abcam, AB_10979735, Cambridge, UK), BCL-2 (1:1500; Abcam, AB_1957474, Cambridge, UK) or GAPDH (1:5000; Abcam, AB_2537658, Cambridge, UK). After washing the membranes with TBST, horseradish peroxidase-conjugated secondary antibody diluted 5000-fold was incubated with the membrane at room temperature for 2 h. Then, the membranes were observed using a Tanon 5200 multifunctional imaging system. GAPDH or ACTIN was used as a loading control.

#### ROS

After treatment, the H9C2 cells were incubated with 10 µM DCFH-DA (Beyotime, Shanghai, China) in the dark at 37 ℃ for 40 min. After washing the cells three times with a serum-free culture medium, images were captured using a fluorescence microscope (Olympus, Tokyo, Japan) and analyzed with Image J.

### Statistical analysis

All data are expressed as the mean ± SEM and were analyzed with SPSS 25.0. All charts were produced by the GraphPad Prism, version 7.0 (GraphPad, San Diego, CA, USA) software. And data was analyzed via one-way analysis of variance (ANOVA) followed by the Bonferroni or Tamhane T2 test. For all tests, differences were considered statistically significant at a level of *p* < 0.05.

## Results

### Hypoxia and reoxygenation decreased viability and increased apoptosis in H9C2 cells

**Fig. 1:**
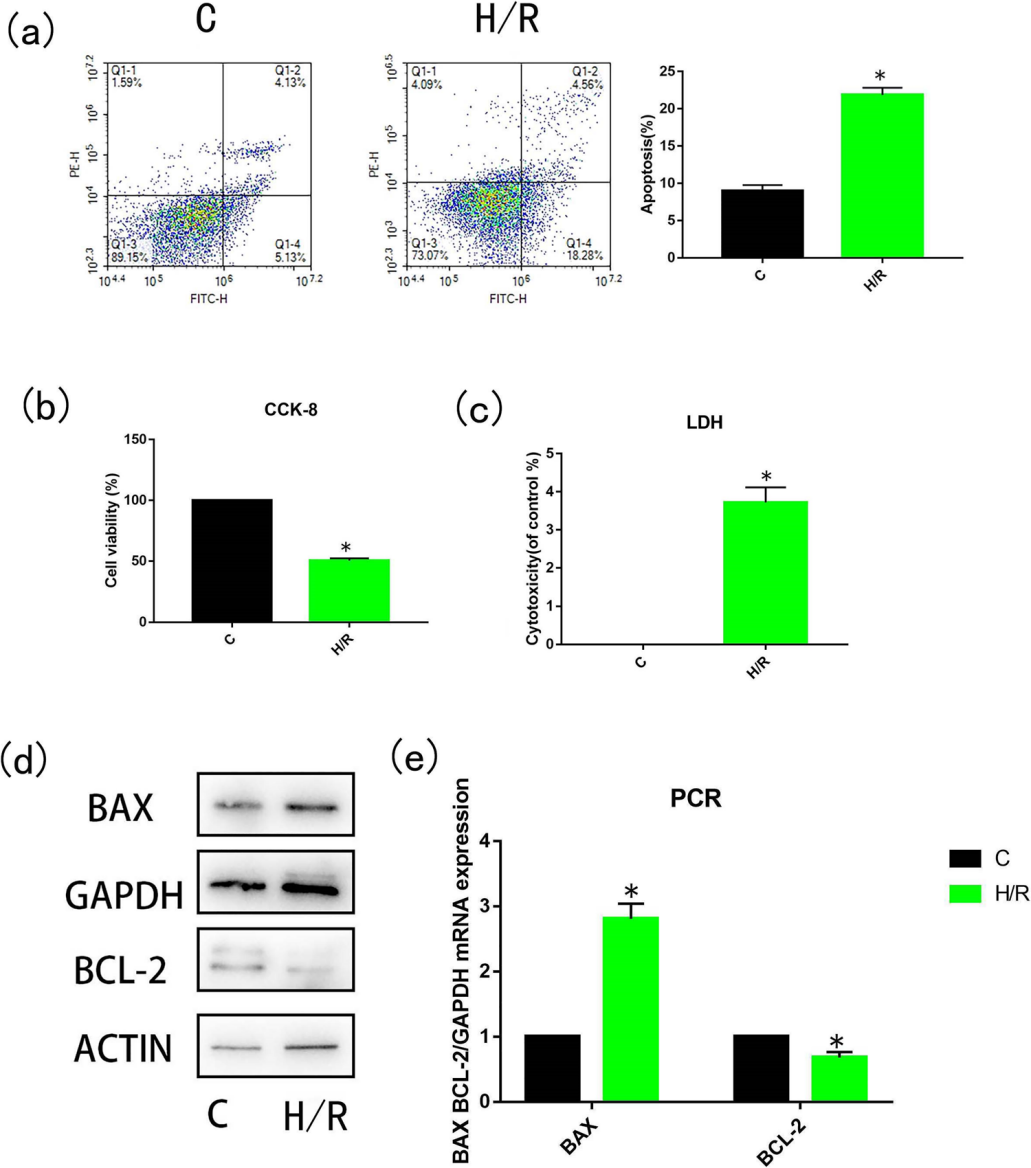
Oxygen–glucose deprivation reduced the viability of H9C2 cells. H9C2 cells were deprived of oxygen and glucose for 6 h (incubated in glucose-free medium without fetal bovine serum, in a 1% O_2_, 5% CO_2_, 94% N_2_, and 37 ℃ environments) and reoxygenated for 4 h (incubated in a high-glucose medium containing fetal bovine serum, in a 5% CO_2_, 37 ℃ environments). Apoptosis rate (**a**), cell viability (**b**), the release of lactate dehydrogenase (**c**), expression of apoptotic proteins Bcl-2 and BAX (**d**), and expression levels of mRNA (**e**) were detected. *N* = 3; *p < 0.05 vs control

In order to verify the effect of H/R on H9C2 cells, we treated the cells with hypoxia for 6 h and reoxygenation for 4 h. Cell apoptosis was detected by flow cytometry. The percentage of apoptosis in the H/R group was significantly higher than that in control group (*p* < 0.05, Fig. 1a). In addition, CCK-8 (Fig. 1b) and LDH (Fig. 1c) assays were used to measure cell viability and membrane integrity, respectively. Western blot and PCR showed that expression of the apoptosis-related proteins Bax was increased in the H/R group, and Bcl-2 was decreased in the H/R group compared with the control group (*p* < 0.05, Fig. 1d, e). Results showed that, compared with the control group, cell viability and membrane integrity were significantly reduced in the H/R group (*p* < 0.05). These data suggest that H9C2 cell viability and apoptosis are affected by H/R.

### Overexpression of HSP70 can reduce apoptosis in H9C2 cells

**Fig. 2:**
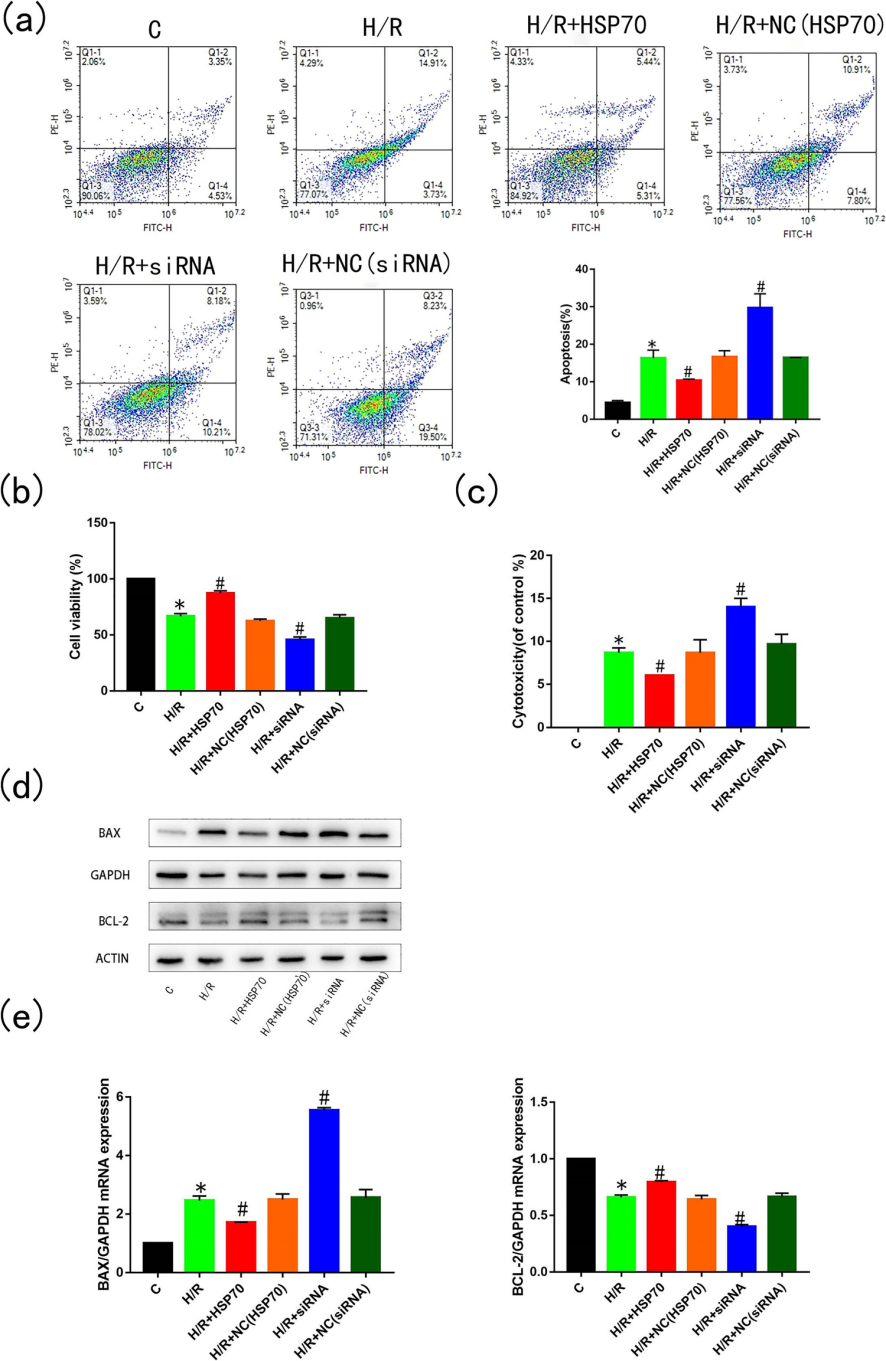
Overexpression of HSP70 can reduce apoptosis in H9C2 cells. Before oxygen and glucose deprivation, H9C2 cells were transfected with a plasmid overexpressing HSP70 (when cells grew to 70% density in a single well of a six-well plate, plasmids, Lipo3000, P3000, and OPTI medium were mixed, added to the cell culture medium and incubated for 6 h) or transfected with siRNA to silence HSP70 (siRNA, Lipo3000 and OPTI medium were mixed and added into the cell culture medium when the cells grew to 70% density in a single well of a six-well plate, and the solution was changed 6 h later). Apoptosis rate (**a**) and cell viability (**b**) were detected after transfection. Lactate dehydrogenase release (**c**), expression of apoptotic proteins Bcl-2 and BAX (**d**) and mRNA expression levels of Bcl-2 and BAX (**e**) were also measured, *n* = 3; *p < 0.05 vs control; #*p* < 0.05 vs HR

H9C2 cells were treated with H/R 24 h after transfection with either amplified HSP70 or siRNA targeting HSP70. Flow cytometry showed that overexpression of HSP70 could significantly reduce the apoptosis rate (*p* < 0.05), whereas silencing of HSP70 could significantly increase the apoptosis rate (*p* < 0.05, Fig. 2a) in H9C2 cells. Similarly, HSP70 overexpression significantly inhibited the decrease in cell viability (*p* < 0.05, Fig. 2b) and significantly increased the membrane integrity (*p* < 0.05, Fig. 2c) in H9C2 cells subjected to H/R. Furthermore, HSP70 overexpression significantly decreased expression of the pro-apoptotic protein BAX (p < 0.05) and increased expression of the anti-apoptotic protein Bcl-2 (*p* < 0.05). When HSP70 was silenced in H9C2 cells, expression of BAX increased (*p* < 0.05) and expression of Bcl-2 decreased (*p* < 0.05, Fig. 2d, e), indicating a switch to pro-apoptotic signaling. These data suggest that HSP70 is involved in H9C2 cell apoptosis induced by H/R and that overexpression of HSP70 can reduce H9C2 cell apoptosis in response to H/R.

### HSP70 can mitigate the increase in intracellular calcium ion and reactive oxygen species concentration induced by hypoxia and reoxygenation through IP3R/STIM1

**Fig. 3:**
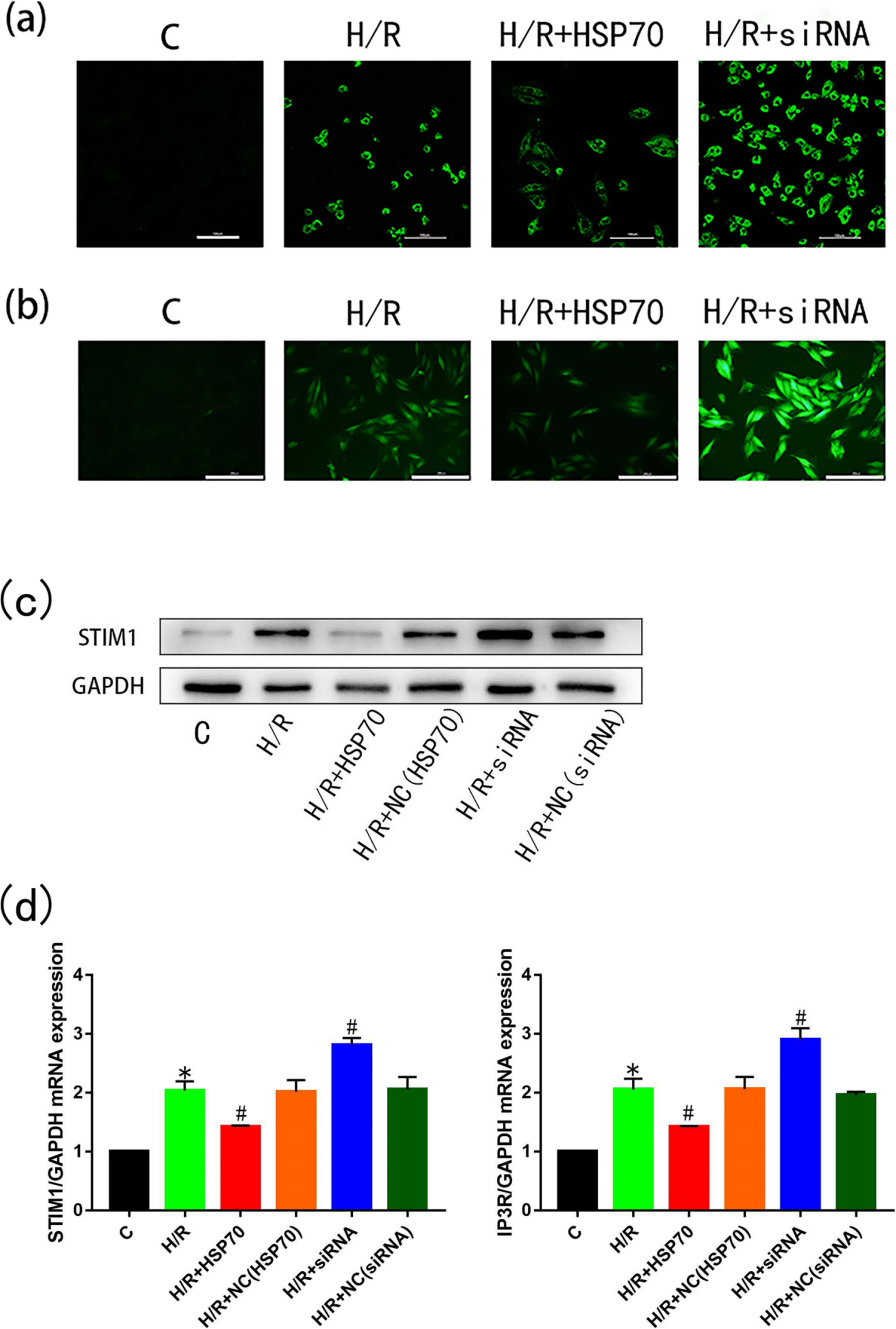
HSP70 can reduce calcium accumulation and ROS production in H9C2 cells treated with oxygen and glucose deprivation. Before oxygen and glucose deprivation, H9C2 cells were transfected with a plasmid overexpressing HSP70 (when cells grew to 70% density in a single well of a six-well plate, plasmids, Lipo3000, P3000, and OPTI medium were mixed, added to the cell culture medium and incubated for 6 h) or transfected with a plasmid expressing anti-HSP70 siRNA (siRNA, Lipo3000, and OPTI medium were mixed, added to the cell culture medium when the cells grew to 70% density in a single well of a six-well plate and incubated for 6 h). The calcium ion content in H9C2 cells was detected by laser confocal microscopy (scale bar: 200 um) (**a**) and ROS content in H9C2 cells was detected by fluorescence microscopy (**b**) (scale bar: 200 um), changes in STIM1 protein expression (**c**) and in IP3R and STIM1 RNA expression (**d**) were measured. *N* = 3; *p < 0.05 vs control; #*p* < 0.05 vs HR

Ca^2+^ overload is an important mechanism of MIRI. To investigate whether overexpression of HSP70 affects intracellular Ca^2+^ accumulation and IP3R and STIM1 were involved in the anti-apoptotic mechanism, the fluorescence intensity of Fluo-3 /AM labeled Ca^2+^ was measured with confocal microscopy after transfection and H/R treatment. We found that, compared with control cells, H/R cells had significantly increased intensity (*p* < 0.05), indicating increased intracellular Ca^2+^ levels. Compared with the untransfected H/R group, the fluorescence intensity was lower after H/R treatment in cells overexpressing HSP70 (*p* < 0.05) but was significantly increased in cells with HSP70 silencing (*p* < 0.05, Fig. 3a). RT-PCR and Western blot showed that IP3R and STIM1 mRNA and protein levels were higher in the H/R-treated cells compared with control cells. Overexpression of HSP70 significantly decreased IP3R and STIM1 expression levels (*p* < 0.05), while silencing of HSP70 further increased IP3R and STIM1 expression levels (*p* < 0.05) in H/R-treated cells (Fig. 3c, d). These data suggest that HSP70 overexpression in H9C2 cells can significantly reduce intracellular Ca^2+^ accumulation after H/R.

Oxidative stress is another central mechanism involved in MIRI. As shown in this result, fluorescence intensity was significantly increased in the H/R group (*p* < 0.05), indicating that ROS level was increased in H9C2 cells after H/R. In addition, compared with untransfected H/R-treated cells, cells overexpressing HSP70 showed significantly reduced fluorescence intensity (*p* < 0.05), while silencing of HSP70 significantly increased the fluorescence intensity (*p* < 0.05, Fig. 3b). These results suggest that overexpression of HSP70 can reduce the calcium ion accumulation and ROS generation seen in response to MIRI.

### Role of STIM1 in apoptosis induced by hypoxia and reoxygenation

**Fig. 4:**
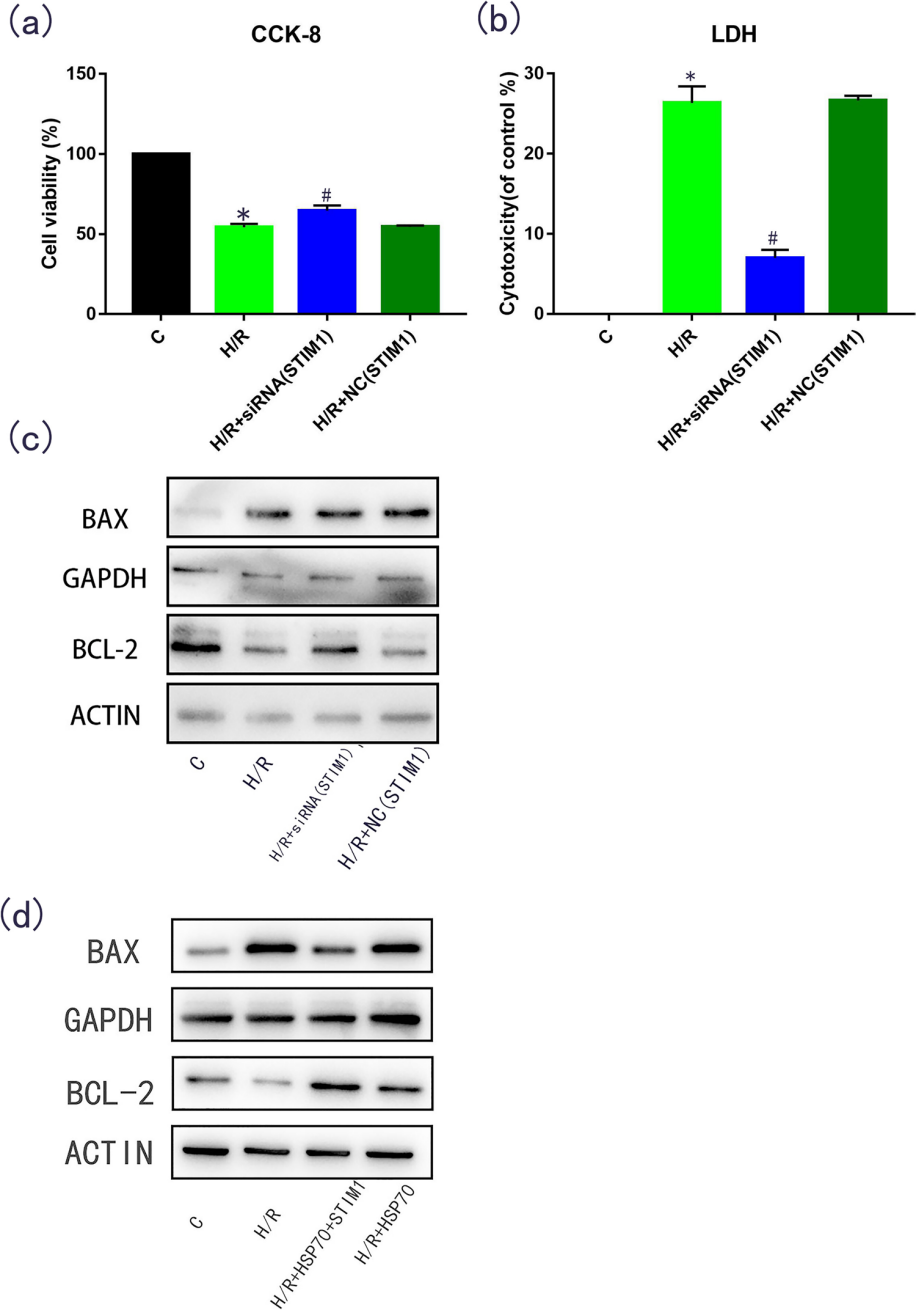
To verify the role of STIM1 in cell apoptosis, siRNA silencing of STIM1 was performed on H9C2 cells (siRNA, Lipo3000, and OPTI medium were mixed, added to the cell culture medium after cell growth to 70% density in a single well of a six-well plate, and incubated for 6 h). H9C2 cell viability (**a**), the release of lactate dehydrogenase (**b**), and expression of apoptotic proteins Bcl-2 and BAX (**c**) were measured in STIM1 knockout H9C2 cells treated with oxygen–glucose deprivation. To verify the role of HSP70-STIM1-apoptosis in cell apoptosis, H9C2 cells with HSP70 overexpression and STIM1 knockout were treated with oxygen and glucose deprivation to detect the expression of apoptotic proteins Bcl-2 and BAX (**d**). *N* = 3; *p < 0.05 vs control; #*p* < 0.05 vs HR

We then silenced STIM1 in H9C2 cells through siRNA transfection to verify the role of STIM1 in H/R. STIM1 silencing led to increased cell viability (*p* < 0.05, Fig. 4a) and decreased LDH release (*p* < 0.05, Fig. 4b). Furthermore, STIM1 silencing decreased the protein and mRNA expression of pro-apoptotic BAX (*p* < 0.05) and increased the protein and mRNA expression of anti-apoptotic Bcl-2 (*p* < 0.05, Fig. 4c).

To further verify the relationship between HSP70-STIM1-apoptosis, we designed a new subgroup, in which one group was simultaneously transfected with the HSP70-overexpressed gene and STIM1-silenced gene. The results showed that compared with the H/R group, the expression of pro-apoptotic protein BAX was significantly decreased (*p* < 0.05, Fig. 4d), and the expression of anti-apoptotic protein Bcl-2 was significantly increased (*p* < 0.05, Fig. 4d). According to the above evidence, we found that STIM1 plays a promoting role in cell apoptosis, and that inhibition of STIM1 expression may lower apoptotic rates in MIRI. Therefore, we can conclude that HSP70 can reduce myocardial apoptosis in the hypoxia and reoxygenation environment by reducing the expression of STIM1.

## Discussion

At present, myocardial ischemia–reperfusion injury is an inevitable clinical problem, which has a great impact on the perioperative period and prognosis of patients. In the past, we have encountered a patient, and the procedure went very smoothly. But a few hours after the patient was transferred back to the intensive care unit, the patient’s heart function dropped sharply, and after a variety of treatments, the patient still died. After investigation, we found that the cause of this situation had little to do with surgical procedures and operations in intensive care units but rather that patients developed myocardial ischemia–reperfusion injuries, which aggravated the already fragile heart function. Myocardial ischemia–reperfusion injuries have always constrained the development of cardiovascular surgery. In the research direction of cardiac surgery, the mechanism of seeking myocardial protection and reducing ischemic reperfusion injury has always been a hot spot and difficult point in its research. Previous studies in our group have confirmed the anti-apoptosis effect of heat shock protein 70 in hypoxic reoxygenation experiments. At the same time, in that study, we found that heat shock protein 70 may have the effect of reducing cytoplasmic calcium ion aggregation and inhibiting calcium overload in cardiomyocytes (Sun et al. 2017). In this study, we explored the protective effect of HSP70 on myocardial cells in an oxygen-glucose deprived environment, providing a new direction for clinical research on myocardial protection.

The main reason is that ischemia is accompanied by the occurrence of hypoxia, resulting in a decrease in ATP hydrolysis in the body and an increase in hydrogen ion production, resulting in the overloading of cellular sodium ions caused by an increase in Na + /H + exchange. Under physiological conditions, reducing sodium ions in the cytoplasm is mainly transported to the cytoplasm by sodium–potassium-ATPase to the cell. However, under ischemic conditions, ATP is depleted and the sodium–potassium-ATPase is inhibited, so the body initiates a Na^+^/Ca^2+^ exchange to reduce sodium ions in the cytoplasm. Extracellular calcium ions are transferred to the cell in large quantities, causing a large aggregation of intracellular calcium ions. Similarly, ATP production is reduced and depleted, and Ca^2+^-ATPase activity in the muscle reticulum is weakened, resulting in impaired calcium transport. The combined effect of the two leads to the occurrence of intracellular calcium overload. Overloading of calcium ions in the cytoplasm activates phospholipase, resulting in an increase in dysfunctional ROS, which in turn leads to oxidative damage to nucleic acids, proteins, and lipids.

HSP70 is an important component of cell homeostasis (Ambrose and Chapman 2021). Under normal circumstances, HSP70 expression in cells is very low. However, when cells encounter environmental changes, they can rapidly increase HSP70 levels to facilitate anti-stress mechanisms (Zatsepina et al. 2021). Moreover, HSP70 is now increasingly being studied in tumor-related fields (Albakova et al. 2020; Murphy 2013; Moradi-Marjaneh et al. 2019). HSP70 provides critical functions that are essential for cancer cell growth and survival (Elmallah et al. 2020). HSP70 is upregulated in most types of cancer (Vostakolaei et al. 2019) and is involved in tumor cell growth, invasion, migration, and resistance to anticancer treatments (Vostakolaei et al. 2021). Based on this study and our previous work, we believe that HSP70 has a good anti-tumor cell death effect that could also function in myocardial protection.

This study showed that glucose deprivation and hypoxia, followed by reoxygenation, significantly decrease the viability of H9C2 cells. H/R also induces LDH release, indicating cytotoxicity, and increases apoptosis in H9C2 cells. Furthermore, HSP70 overexpression in H9C2 cells mitigated the changes induced by H/R. Cells overexpressing HSP70 showed significantly higher cell viability, decreased LDH release, and less cell apoptosis in response to H/R. This suggests that HSP70 has a protective effect on the damage caused by oxygen and glucose deprivation in H9C2 cells. In addition, we found a negative correlation between HSP70 and IP3R/STIM1 expression. We also verified that silencing STIM1 can alleviate the damage in H9C2 cells caused by oxygen and glucose deprivation. Therefore, STIM1/IP3R may be a key factor in H9C2 cell damage and apoptosis after H/R. HSP70 is expressed at low levels in normal cells. When cells are subjected to high temperatures or other harmful stress conditions, the HSP70 expression level rapidly increases in a short period of time to facilitate the cellular anti-stress response (Clerico et al. 2019). Therefore, we used transfection to overexpress HSP70 in normal H9C2 cells to explore the effect of HSP70 on H/R-induced myocardial cell damage. We found that overexpression of HSP70 mitigated the decreased cell viability and increased cell apoptosis seen in cells subjected to H/R. The Bcl-2 family of proteins are key regulators of apoptosis and include the pro-apoptotic BAX protein and the anti-apoptotic Bcl-2 protein. Down-regulation of BAX and up-regulation of Bcl-2 can reduce apoptosis. In this study, we demonstrated that HSP70 expression can downregulate BAX, upregulate Bcl-2 and reduce cell apoptosis. The opposite effect was observed when HSP70 was silenced. In summary, these data suggest that HSP70 has a protective effect against cell damage caused by glucose deprivation and H/R.

Calcium overload plays an important role in MIRI. A high concentration of intracellular calcium ions can lead to apoptosis. Therefore, finding ways to inhibit calcium overload is key to preventing MIRI. IP3R and STIM1 are both important intracellular calcium channels that mediate intracellular calcium changes. STIM1 plays an important role in apoptosis in H9C2 cells (He et al. 2017), but as far as we know, the question of whether HSP70 can affect IP3R and STIM1 expression has still not been explored. Our current study confirmed that HSP70 overexpression can reduce IP3R and STIM1 expression in response to glucose deprivation and H/R. In contrast, HSP70 silencing can increase the expression levels of IP3R and STIM1 in response to glucose deprivation and H/R. Finally, we showed that inhibiting STIM1 expression can reduce apoptosis in H9C2 cells. These results suggest that HSP70 may inhibit the expression of IP3R and STIM1, thus playing an anti-apoptotic role by preventing intracellular calcium overload.

Nevertheless, our study has some limitations. We did not use relevant animal experiments to verify whether specific I/R injury can be altered in vivo by HSP70, IP3R, and STIM1. Therefore, the clinical relevance of these results is not clear. We will focus on this aspect in a follow-up in vivo study.

## Conclusion

HSP70 may play a role in myocardial protection against MIRI by regulating IP3R/STIM1 expression and thus alleviating intracellular calcium overload, and by lowering expression of the pro-apoptotic protein BAX, thus reducing apoptosis in H9C2 cells. Our study demonstrated that HSP70 has an anti-apoptotic effect on myocardial cells, providing a potential new therapeutic mechanism for clinical protection against MIRI.
